# Advanced Wilms Tumor in a Middle‐Aged Adult: A Case Report

**DOI:** 10.1002/ccr3.71577

**Published:** 2025-11-30

**Authors:** Denis Mucunguzi, Bartholomeo Nicholaus Ngowi, Orgeness Jasper Mbwambo, Phinihas Jackson Mwijumbe, Nyamhanga Nsaho Maro, Abitalis Mayengela, Angumbwike Mwakitwange, Patrick Amsi, Donald Dominick Lema, Alex Mremi

**Affiliations:** ^1^ Department of Urology Kilimanjaro Christian Medical Centre Moshi Tanzania; ^2^ School of Medicine KCMC University Moshi Tanzania; ^3^ Division of Urology Mbarara University of Science and Technology Mbarara Uganda; ^4^ Department of Pathology Kilimanjaro Christian Medical Centre Moshi Tanzania; ^5^ Department of Research and Training Kilimanjaro Clinical Research Institute Moshi Tanzania

**Keywords:** case report, middle‐aged adult, nephroblastoma, renal neoplasm, Wilms tumor

## Abstract

Wilms tumor, or nephroblastoma, is a malignant embryonal tumor originating from nephrogenic blastema, which imitates the histology of a developing kidney. Primarily, it occurs in children. Wilms tumor is exceedingly rare in adults, where the diagnosis is often delayed, and the prognosis tends to be worse compared to children. Owing to their rarity in the adult population, Wilms tumors do not have true treatment guidelines and are instead treated with regimens identical to those for pediatric tumors. Herein, we present a case of Wilms tumor in a middle‐aged adult and review the relevant literature. A 48‐year‐old female presented to our facility with a long‐standing history of left flank pain for more than 4 months. Upon examination, a huge palpable mass in the left lumbar region was noted. An abdominal‐pelvic CT scan revealed a large, complex, heterogeneous, enhancing cortical renal mass with cystic and solid components. The mass had an exophytic growth, surrounded by fat tissue, occupying the lower pole of the left kidney, compressing renal parenchyma with no infiltration of the renal vein or inferior vena cava. A clinical diagnosis of renal cell carcinoma was considered, and surgery was recommended. Pathology evaluation of the nephrectomy specimen confirmed it to be a Wilms tumor. She was discussed in a multidisciplinary team, and a consensual decision was made to be given palliative chemotherapy with a combination of vincristine, dactinomycin, doxorubicin, and cyclophosphamide after optimization. This case report highlights the rarity of Wilms tumor in adults, especially in middle age, and emphasizes the importance of considering it in the differential diagnosis of renal masses.

## Key Clinical Message

Wilms tumor is the most common renal malignancy in children, but very rare in adults. Adult diagnosis is difficult due to nonspecific imaging and similarity to other renal tumors. Histopathology is identical in adults and children, and treatment follows pediatric protocols due to a lack of adult‐specific guidelines.

## 1 Introduction

Nephroblastoma, also known as Wilms tumor, is the most common form of kidney cancer in children, representing approximately 7% of all pediatric malignancies and the vast majority of renal tumors in this age group [1]. The tumor typically presents in early childhood, with a peak between 3 and 4 years of age. Conversely, in adults, the occurrence of Wilms tumor is exceedingly rare, at just 0.2 cases per million per year [2]. Wilms tumor arises from nephrogenic blastemal cells and displays diverse patterns of differentiation that resemble developing kidney tissue [3]. While most cases occur sporadically, about 10% are associated with congenital anomalies or syndromic conditions such as Beckwith–Wiedemann, Denys–Drash, WAGR, and Perlman syndromes [1, 4]. Mutations in the tumor suppressor genes WT1 and WT2, which are found on chromosomes 11p13 and 11p15, respectively, have been linked to a genetic susceptibility to Wilms tumor [3, 4].

The challenges associated with adult Wilms tumor are its aggressiveness, rarity, and lack of established treatment protocols [1]. In their study, He et al. (2025) highlighted the low preoperative diagnostic accuracy and poorer prognosis of adult Wilms' tumor, reporting a 5‐year progression‐free survival of 50% compared with that in children [5]. Consistent with these findings, Fernández‐Ferreira et al. [6] and Modi et al. [7] highlight that treatment guidelines remain undefined and outcomes in adults are generally worse than in children. The clinical manifestations of adult Wilms tumor include hematuria and abdominal pain, and it is not uncommon for patients to have metastasis to the liver and lungs [8]. Adult Wilms tumor should be differentiated from renal cell carcinoma (RCC) because, although they can appear similar radiologically and histologically, they differ in origin, treatment approach, and prognosis [7, 9]. Accurate diagnosis is essential to avoid mistreatment and ensure appropriate multimodal therapy. Rarely, Wilms' tumor and RCC can be found simultaneously in the same kidney [10].

Despite the fact that many regimens have been established for the treatment of pediatric Wilms tumor, there are no definitive guidelines for the adult form of this malignancy [11]. The Children's Oncology Group (COG) and the International Society of Pediatric Oncology (SIOP) have undertaken studies and trials for children that serve as the foundation for current treatments [12]. The modified pediatric protocols are applied to adult Wilms tumor [11]. Adult patients with high‐risk disease or unfavorable histology have a poor prognosis even with extensive multimodal therapy, and some authors have suggested an aggressive treatment irrespective of stage [12]. Although Wilms tumor was not suspected preoperatively, the surgical resection and subsequent multimodal therapy coincidentally aligned with the principles of the National Wilms Tumor Study (NWTS) treatment concept [12, 13]. This case report aims to provide information on an adult patient who was diagnosed with Wilms tumor, focusing on diagnosis and therapeutic strategies for this rare condition.

## 2 Case History/Examination

We report a 48‐year‐old female who presented to our hospital with a long‐standing history of left flank pain for more than 4 months, radiating to the lower back and extending to the lumbar region, associated with abdominal fullness and early satiety as well as headaches and generalized body weakness, but reported no history of fevers, unintentional weight loss, hematuria, or lower urinary tract symptoms. Review of other systems was unremarkable. This was the index admission, which reported no history of any surgeries, food or drug allergies, or blood transfusions. Reported history of taking alcohol for 20 years, no history of smoking, and no history of hereditary diseases in the family.

On physical examination, she was fully conscious, afebrile with mild conjunctival pallor, not jaundiced, with a blood pressure of 150/88 mmHg, a pulse rate of 70 beats per minute, and a respiratory rate of 16 breaths per minute. The abdomen was of normal fullness, moving well with respiration, with no visible scars, soft with no tenderness; however, there was a palpably enlarged mass in the left lumbar region measuring about 15 by 15 cm, firm, and dull on percussion. There were no other palpably enlarged masses; the percussion note was tympanic, and the bowel sounds were present, with about three bowel sounds per minute. Other systemic examinations were normal.

## 3 Differential Diagnosis, Investigations, and Treatment

**FIGURE 1:**
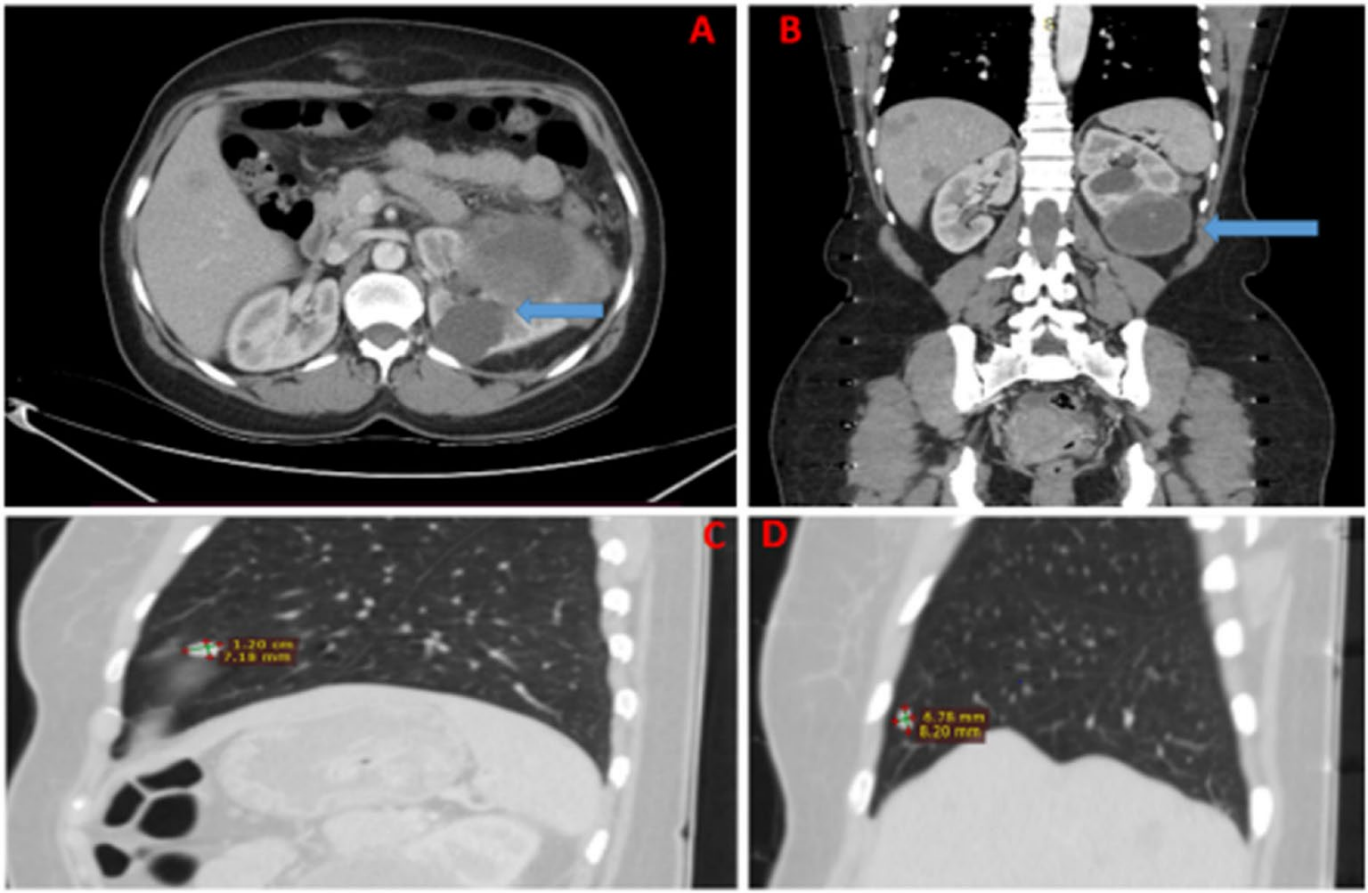
Axial abdominal pelvic CT image (arterial phase) showing a large cortical complex heterogenous enhancing renal mass with cystic and solid components (blue arrow) (A); coronal view of the abdominal pelvic CT scan shows the left renal tumor with solid and cystic components marked on the lower pole. Cysts are seen in the right kidney. Hypodense lesions seen in the liver are suggestive of possible metastasis or hepatic cysts (blue arrow) (B); sagittal CT scan views of the left lung (C) and right lung (D) demonstrate nodules suggestive of metastasis, respectively.

Her laboratory blood workup was essentially normal, except for deranged serum creatinine. According to the SIOP‐RTSG 2016 Umbrella protocol, MRI is recommended as the primary diagnostic modality for Wilms tumor; however, in the present case [16], CT was performed due to limited access to MRI, which nevertheless allowed for adequate preoperative assessment. The contrast‐enhanced abdominal‐pelvic CT scan revealed a large, complex, heterogeneous, enhancing cortical renal mass with cystic and solid components, growing exophytic and surrounding fat stranding, occupying the lower pole of the left kidney, compressing renal parenchyma with no infiltration of the renal vein or inferior vena cava thrombus (Figure 1A). An enlarged liver measuring 17.5 cm with hypodense lesions was seen, suggestive of possible metastasis. Multiple cysts were seen in both kidneys (Figure 1B). The exposed chest showed two subcentimeter suspicious pulmonary nodules, one in the right anterior basal segment (6.9 mm) and another one noted in the left mediastinum subpleural base (1.1 cm). Suspicious pulmonary metastases were noted (Figure 1C,D). A working clinical diagnosis of a left complex renal tumor with differentials of renal cell carcinoma, malignant renal cysts, secondary malignancy of the left kidney, and multiple liver cysts was considered. Upfront surgery was recommended by a panel of specialist urologists.

**FIGURE 2:**
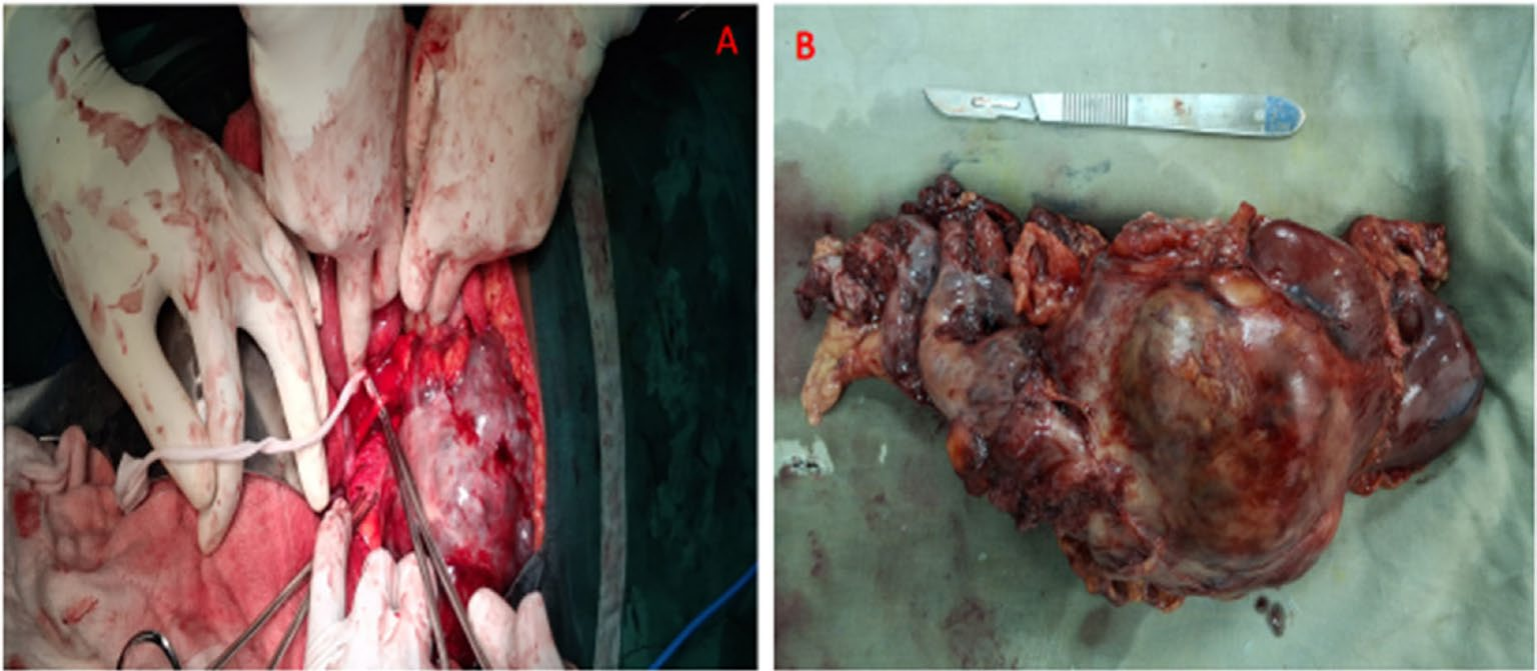
An intraoperative photograph shows the left ureter and an irregular, multinodular hemorrhagic left renal tumor in situ at the exploratory laparotomy surgical procedure (A); a resected left renal specimen with a fixed hemorrhagic tumor (B).

After obtaining the patient's informed consent for surgical management, surgery was scheduled and performed under general anesthesia under aseptic conditions. A left transverse anterior subcostal incision was made, and the abdomen was opened in layers to access the peritoneal cavity. Hemorrhagic ascites and seeding of tumor around the omentum were seen, and a left irregular hemorrhagic renal complex mass with solid and cystic components was seen occupying the lower pole (Figure 2A). There were no enlarged nodes. A radical left nephrectomy procedure was performed by a team of expert urological surgeons. A resected specimen (Figure 2B) was submitted for histopathology examination. Postoperative care was uneventful. The patient was kept on antibiotics, maintenance fluids, and comprehensive nursing care. She was discharged 3 days after the procedure. During a subsequent follow‐up visit, the surgical site incision had healed completely, and her performance status was ECOG 1.

## 4 Conclusion and Results (Outcome and Follow‐Up)

**FIGURE 3:**
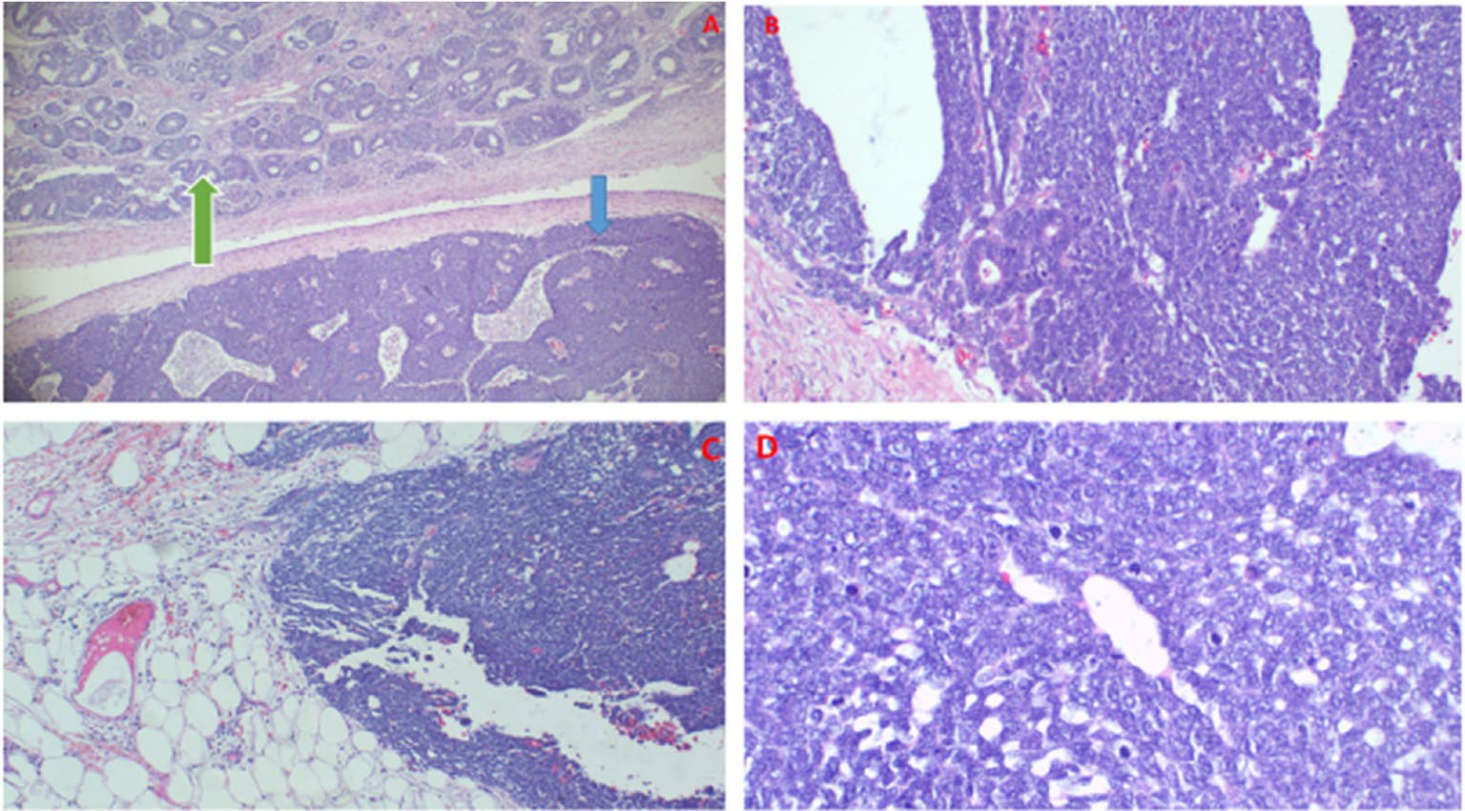
Histopathology of Wilms tumor demonstrates a biphasic tumor composed of hyperchromatic, small undifferentiated cells (blastema) highlighting a serpentine growth pattern (blue arrow) and an epithelial component forming tubules or poorly formed rosettes (green arrow); hematoxylin and eosin (H&E) staining at 2 × original magnification (A); a blastema component showing a diffuse growth pattern, H&E staining at 4 × original magnification (B); extra‐capsular tumor extension to peri‐renal fat invasion (C); and diffuse anaplasia displaying marked atypical cells with frequent mitoses, H&E staining at 10 × original magnification (D).

**FIGURE 4:**
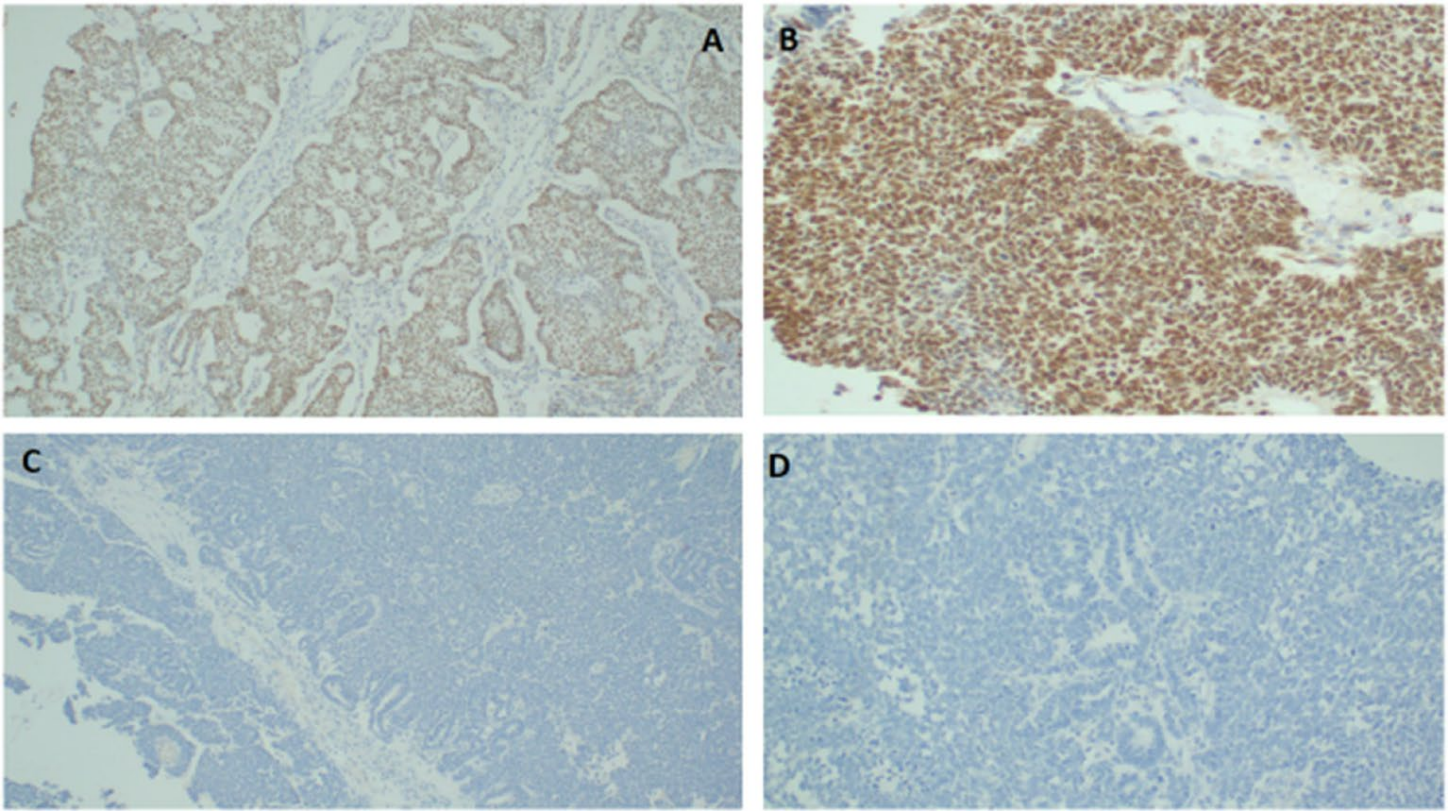
Immunohistochemical staining of Wilms tumor highlights strong immunostaining of the epithelial cell component with cytokeratin (A), strong immunostaining of blastema and epithelial components with WT1 (B), negative immunostaining of the tumor cells with synaptophysin (C), and cytokeratin 7 (D).

The final histopathology report described an infiltrative tumor comprised of two distinct cell components: blastema—small‐ to medium‐sized undifferentiated cells with small nuclei, showing serpentine and diffuse growth with areas of diffuse cellular anaplasia. Elsewhere, the epithelial cell component was represented by atypical tubules or rosette‐like structure formation (Figure 3A–D). Immunohistochemical test results highlighted strong immunostaining of the tumor cells with WT1 but were negative for cytokeratin and synaptophysin (Figure 4A–D). The findings were consistent with Wilms tumor, high risk, stage III, with blastemal predominant. The patient was sent to the oncology department for further adjuvant treatment.

## 5 Discussion

Nephroblastoma, also known as a Wilms tumor, is a very uncommon cancer in adults, occurring in less than 1% of cases, but it makes up 5% of childhood malignancies and affects roughly 1 in 10,000 children [1]. In adults, fewer than 300 cases have been reported, which poses serious challenges for diagnosis and treatment and frequently results in delayed or inaccurate diagnoses [6].

In adults, Wilms tumor can closely resemble RCC, which accounts for about 90%–95% of adult renal malignancies [9]. Typical symptoms include flank pain, hematuria, weight loss, and reduced performance status [7], whereas pediatric cases more often present with a painless, rapidly enlarging abdominal mass. On examination, left‐sided tumors may mimic splenomegaly and right‐sided ones hepatomegaly. Rare manifestations include varicocele, hernia, testicular enlargement, cardiac failure, hypoglycemia, Cushing syndrome, pleural effusion, and acute abdominal complications such as rupture or hemorrhage [12]. Adult Wilms tumor generally follows a more aggressive course with higher recurrence rates and limited therapeutic options [8].

The initial evaluation and workup consist of baseline investigation as well as imaging studies. Although CT scans remain useful, recent SIOP‐RTSG (UMB RELLA 2016) and contemporary imaging reviews recommend contrast‐enhanced MRI as the preferred modality for local staging and characterization of renal (Wilms) tumors, because MRI gives superior soft‐tissue contrast and better assessment of tumor extent, renal parenchymal involvement, nephrogenic rests/bilateral disease, and response to therapy. In adults with suspected Wilms tumor, there are no widely accepted adult‐only imaging guidelines; most groups therefore apply the pediatric imaging standards (MRI‐first approach) while adapting for adult anatomy and local resource availability [14]. MRI scans are used to evaluate tumor size, characterization, lymphadenopathy, evidence of vascular involvement, and metastases.

As evidenced in our patient, the management of Wilms tumor in adults poses significant challenges in low‐resource settings. Diagnosis is often delayed or mistaken for RCC due to its rarity in adults and the limited availability of advanced imaging and immunohistochemistry [9]. Optimal treatment requires multimodal therapy, yet access to specialized oncologic surgeons, chemotherapeutic agents, and radiotherapy facilities is frequently restricted [7]. Even when chemotherapy is available, treatment interruptions and inadequate supportive care remain common. Financial constraints, out‐of‐pocket costs, and poor follow‐up adherence further compromise outcomes, as many patients present late or default after partial treatment. Moreover, the absence of adult‐specific treatment guidelines, with most regimens being extrapolated from pediatric protocols, compounds uncertainty in management [6, 7].

These barriers highlight the urgent need for improved diagnostic infrastructure, sustainable drug supply, and context‐adapted treatment strategies to optimize care for adult Wilms tumor patients in resource‐limited environments. To address these barriers, strengthening diagnostic infrastructure, ensuring sustainable access to essential chemotherapeutic agents, and training multidisciplinary oncology teams is critical. Adaptation of pediatric treatment protocols for adult use, regional collaborations to share expertise, and the use of tele‐oncology for case discussions may also help optimize care [6]. Establishing affordable follow‐up programs and patient support systems can improve adherence and long‐term outcomes in resource‐limited environments.

In our case, an abdominal ultrasound scan revealed a large heterogeneous and hypoechoic mass involving the left kidney. A contrast‐enhanced abdominal‐pelvic CT scan revealed a large, heterogeneous, contrast‐enhanced cortical complex left renal mass with cystic and solid components, growing exophytically and surrounded by fat stranding, occupying the lower pole of the left kidney. In adults, the scarcity of cases and nonspecific clinical and radiologic findings commonly result in delayed diagnosis or misclassification as RCC [15]. There is no standard treatment for adult Wilms tumor; however, it is recommended to treat based on the National Wilms' Tumor Study Group (NWTS) [12]. A histopathological examination is often necessary to confirm the diagnosis. Microscopically, the tumor typically exhibits a triphasic morphology consisting of stromal, epithelial, and blastemal components. In our case, the tumor demonstrated a biphasic pattern with a predominance of blastemal over epithelial components.

Recent reviews have reported close to 300 adult cases of Wilms tumor described in the literature [6]. This highlights the rarity of the disease and the need for further collaborative research to improve understanding and management. Although the overall survival rate for children with Wilms tumor exceeds 90%, prognosis is influenced by age at diagnosis. Younger children generally experience more favorable outcomes, whereas children older than 4 years show a less favorable prognosis [14].

## 6 Conclusion

Wilms tumor in adults is a rare entity, and its diagnosis is generally made on postoperative histopathological examination. Due to the rarity of Wilms' tumor in adults, there is no standard treatment, and this poses a risk of receiving inadequate or inappropriate therapy. It has a worse prognosis than pediatric Wilms tumor.

## Author Contributions

**Denis Mucunguzi:** conceptualization, project administration, resources, writing – original draft, writing – review and editing. **Bartholomeo Nicholaus Ngowi:** conceptualization, supervision, writing – review and editing. **Orgeness Jasper Mbwambo:** resources, writing – review and editing. **Phinihas Jackson Mwijumbe:** resources, writing – review and editing. **Nyamhanga Nsaho Maro:** resources, supervision, writing – review and editing. **Abitalis Mayengela:** writing – review and editing. **Angumbwike Mwakitwange:** writing – review and editing. **Patrick Amsi:** investigation, validation, visualization. **Donald Dominick Lema:** supervision, writing – review and editing. **Alex Mremi:** investigation, validation, visualization, writing – review and editing.

## Funding

The authors have nothing to report.

## Ethics Statement

We obtained a waiver from the KCMC University Institutional Review Board.

## Consent

Written informed consent was obtained from the patient for the publication of this case report and accompanying images. A copy of the written consent is available for review by the editor‐in‐chief of this journal upon request.

## Conflicts of Interest

The authors declare no conflicts of interest.

## Data Availability

The data that support the findings of this study are available on request from the corresponding author. The data are not publicly available due to privacy or ethical restrictions.
